# The role of autophagy in SIM mediated anti‐inflammatory osteoclastogenesis through NLRP3 signaling pathway

**DOI:** 10.1002/iid3.1145

**Published:** 2024-01-10

**Authors:** Yuting Cheng, Wenjun Jin, Lin Zheng, Xiaolin Huang, Shanshan Luo, Wei Hong, Jian Liao, Bancha Samruajbenjakun, Chidchanok Leethanakul

**Affiliations:** ^1^ Faculty of Dentistry Prince of Songkla University Hat Yai Thailand; ^2^ School/Hospital of Stomatology Guizhou Medical University Guiyang China; ^3^ Hospital of Stomatology Zhongshan Guangdong China; ^4^ Key Laboratory of Endemic and Ethnic Diseases, Ministry of Education Guizhou Medical University Guiyang China

**Keywords:** autophagy, inflammatory bone resorption, NLRP3, osteoclast, Simvastatin

## Abstract

**Background:**

Inflammatory bone resorption is a prominent risk factor for implantation failure. Simvastatin (SIM) has anti‐inflammatory effects independent of cholesterol lowering and reduces osteoclastogenesis by decreasing both the number and activity of osteoclasts. However, the specific mechanism of inflammatory bone loss alleviation by SIM remains to be elucidated. We hypothesized that SIM relieves inflammatory bone loss by modulating autophagy and suppressing the NOD‐like receptor family pyrin domain‐containing protein 3 (NLRP3) signaling pathway.

**Methods and results:**

RAW264.7 cells were stimulated by lipopolysaccharide (LPS) after being pretreated with various concentrations of SIM. Osteoclast (OC) differentiation, formation and activity were evaluated by tartrate‐resistant acid phosphatase staining, F‐actin ring staining and bone resorption pit assays, respectively. We observed autophagosomes by transmission electron microscopy. Then NLRP3 inhibitor MCC950 was used to further explore the corresponding molecular mechanism underlying anti‑inflammatory bone resorption, the expression of autophagy‐related proteins and NLRP3 signaling pathway factors in pre‐OCs were evaluated by western blot analysis, and the expression of OC‑specific molecules was analyzed using reverse transcription‑quantitative polymerase chain reaction. The results showed that SIM decreased the expression of tumor necrosis factor‐α, whereas increased Interleukin‐10. In addition, SIM inhibited LPS‐induced OC differentiation, formation, bone resorption activity, the level of autophagosomes, and OC‑specific markers. Furthermore, SIM significantly suppressed autophagy by downregulating LC3II, Beclin1, ATG7, and NLRP3‐related proteins expression while upregulating P62 under inflammatory conditions.

**Conclusions:**

SIM may reduce autophagy secretion to attenuate LPS‐induced osteoclastogenesis and the NLRP3 signaling pathway participates in this process, thus providing theoretical basis for the application of this drug in peri‐implantitis.

## Graphical abstract

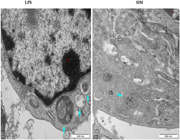

## 1 INTRODUCTION

In recent years, studies have shown that peri‐implantitis has become a serious problem with the increasing number of implants each year.^1^, ^2^ Peri‐implantitis is one of the main complications of implant surgery and an important reason for the failure of implant repair treatment.^3^, ^4^ A cross‐sectional study showed that peri‐implant diseases were very common in dental implant patients, among whom the prevalence of peri‐implantitis exceeded 50%.^5^ Peri‐implantitis is characterized by inflammatory lesions of the peri‐implant, resulting in a progressive loss of supporting bone tissue, which causes the implant to eventually loosen and fall out.^6^ Therefore, inflammatory bone boss around the implant is the key factor affecting the long‐term stability and survival rate of implant dentures.^7^ A favorable local bone microenvironment is beneficial to promote the osseointegration of the implant.^8^ However, persistent inflammatory responses could be caused by infectious bone diseases. Furthermore, the affected sites may be secondary to local metabolic disorders, destruction, and bone death.^9^ Studies have shown that chronic bone metabolic diseases caused by bone trauma and infection have been linked to inflammatory mediators and autophagy, which both play important roles in the regulation of homeostasis in the bone immune system.^10^, ^11^

The NOD‐like receptor family pyrin domain‐containing protein 3 (NLRP3) inflammasome is the first line of defense against pathogens. The NLRP3 inflammasome is activated by pathogen‐associated molecular patterns or damage‐associated molecular patterns and activates the nuclear factor κB (NF‐κB) signaling pathway through the Toll‐like receptor pathway and further stimulates NLRP3 inflammasome assembly and activation. ASC is an adapter molecule that connects NLRP3 and caspase‐1 precursors and then recruits the precursor caspase‐1 into an activated form. Activated caspase‐1 cleaves the precursor of Interleukin‐1β (IL‐1β) into its mature form and causes inflammatory bone resorption, ultimately leading to bone loss.^10^, ^12^, ^13^, ^14^ Moreover, a highly active NLRP3 inflammasome can enhance the bone resorption capacity of OCs by restructuring the actin cytoskeleton.^15^ Simvastatin (SIM), an inhibitor of 3‐hydroxy‐3‐methylglutaryl coenzyme A reductase, is drug for lowering serum cholesterol.^16^, ^17^ In addition to lowering cholesterol, studies have well established that statins also have anti‐inflammatory properties and suppress osteoclastic differentiation in bone tissue.^18^, ^19^ SIM can effectively promote the osseointegration of implants in animal study.^20^ However, the mechanism by which SIM inhibits the differentiation and activity of OCs by regulating NLRP3 signaling pathway remains to be further investigated.

As a cellular stress response mechanism, autophagy plays an immunomodulatory role by regulating microbial invasion and controlling the secretion of immune signaling molecules and inflammatory mediators.^20^ The interaction between autophagy and inflammatory mediators has become a research hotspot. The activation of the NLRP3 inflammasome can regulate the induction of autophagy, while autophagy can regulate the activation of the inflammasome and inhibit its activity.^21^, ^22^ Moreover, studies have shown that appropriate autophagy has an inhibitory effect on the NLRP3 inflammasome.^23^, ^24^ In the inflammatory bone microenvironment, activation of the NLRP3 inflammasome leads to overactivation of OC autophagy. This consequently leads to an increase in the number and activity of OCs, disrupts bone homeostasis, and ultimately results in inflammatory bone resorption.^25^, ^26^ Lipopolysaccharide (LPS), which is a major inflammatory component of the outer membrane of Gram‐negative bacteria, can induce OC formation independent of RANKL.^27^, ^28^ Our recent preliminary experiments showed that the expression of NLRP3 inflammation‐related proteins and autophagy‐related proteins in OCs treated with LPS was significantly increased, and related inflammatory factors and autophagy‐related proteins were significantly decreased after treatment with SIM. The number and activity of OCs also decreased, thereby inhibiting bone resorption induced by LPS. We hypothesized that SIM regulates the NLRP3 signaling pathway by regulating the autophagy level of OCs and thus changes the function and number of OCs to alleviate bone resorption in peri‐implantitis. To date, no study has reported on this topic.

Therefore, this study proposes the following scientific hypothesis: SIM regulates the NLRP3 signaling pathway by reducing the level of overactivated autophagy in inflammatory bone diseases, thereby inhibiting the differentiation and maturation of OCs and delaying the occurrence and development of inflammatory bone resorption in peri‐implantitis.

## 2 MATERIALS AND METHODS

### 2.1 Cells, reagents and antibodies

The RAW264.7 mouse macrophage (Cat#TIB71) cell line was obtained from the American Type Culture Collection. SIM (Cat#PHR1438‑1G) and LPS (Cat#L2630‐10MG) were purchased from Sigma‑Aldrich, Merck KGaA. MCC950 (CP‐456773, Cat# GC31644) was purchased from Glpbio Technology. Fetal bovine serum (FBS, Cat#10099141C) and the α‐modification of Eagle's medium (α‐MEM, Cat#C12571500BT) were purchased from Gibco, Thermo Fisher Scientific, Inc. A tartrate‐resistant acid phosphatase (TRAP) staining kit (Cat#G1492), and 4′,6‐diamidino‐2‐phenylindole (DAPI) and TRITC phalloidin (Cat#CA1610) were purchased from Beijing Solarbio. Bone slice (Cat#Q2356) was purchased from Shanghai Millennium Biology. Specific antibodies against NLRP3 (Cat#23094‐1), ATG7 (Cat#6251), P62 (Cat#4844), Beclin‐1(Cat#19662), caspase‐1 (Cat#16883), tumor necrosis factor‐α (TNF‐α) (Cat#19147), LC3II (Cat#18709) were obtained from Abcam; cleaved‐caspase‐1 (Cat#89332) and IL‐1β (Cat#12507) were obtained from Cell Signaling Technology. Interleukin‐10 (IL‐10) (Cat#60269‐1‐lg). horseradish peroxidase (HRP)‐conjugated goat anti‐rabbit IgG secondary antibody (Cat#L3012) was purchased from Signalway Antibody. Polyvinylidene difluoride (PVDF, ISEQ. 00010) membranes were obtained from EMD Millipore.

### 2.2 Cell culture

RAW264.7 cells were cultured in α‐MEM containing 1% penicillin‒streptomycin and 10% FBS at 37°C in a humidified atmosphere of 5% CO_2_. Nonadherent cells were discarded by changing the medium every 72 h. For experiments, cells were seeded on 6‐ or 24‐, or 96‐well plates in α‐MEM and cultured overnight to allow the cells to attach to the surface. After 24 h, the culture medium was replaced with medium containing LPS (100 ng/ml) and SIM.

### 2.3 In vitro osteoclastogenesis assays

RAW264.7 cells were plated in 24‐well plates (1 × 10^4^ cells per well) or 96‐well plates (1.5 × 10^3^ cells per well) with or without LPS (100 ng/mL) and 1 µM SIM for 3 days or 10 days at 37°C. The cell culture medium was replaced with fresh medium every 2 days.

### 2.4 TRAP staining

The medium was removed and the cells were washed three times with phosphate‐buffered saline (PBS) (shaking gently) on the third day. The cells were fixed with 4% paraformaldehyde for 20 min at 4°C and washed for 10 min with shaking three times. The cells were stained for TRAP using a commercially available kit according to the manufacturer's protocol for 4 h at 37°C in the dark. Cells that stained dark red with three or more nuclei were counted as OCs under the microscope (Leica)

### 2.5 F‐actin ring staining

To detect the formation of F‐actin rings and nuclei, the cells were stained with TRITC phalloidin and DAPI. RAW264.7 cells were cultured on circular microscope cover glass in 24‐well plates. On the third day of induction, the cells were washed twice with PBS, fixed with 4% formaldehyde for 15 min at room temperature, and then washed three times in PBS. To increase permeability, 0.25% Triton X‐100 was added and incubated for 6 min, and the samples were washed three times with PBS. F‐actin rings were stained with TRITC rhodamine‐conjugated TRITC phalloidin for 30 min and the nuclei were stained with DAPI at room temperature for 30 s. Finally, the cells were rinsed extensively with PBS three times, visualized and quantified with a fluorescence microscope (Olympus).

### 2.6 Bone resorption pit assay

The resorptive function of the mature OCs derived from LPS‐stimulated RAW264.7 cells was analyzed on sterile bovine bone slices, which were placed in 96‐well plates with three replicates for each condition. The cells were removed from the bone slices by PBS on the 10th day, and the resorption pits were then visualized under a scanning electron microscope (Hitachi). The total number and area of the resorption pits were quantified and compared using ImageJ software 6.0.

### 2.7 Transmission electron microscopy (TEM)

The autophagosomes were observed by TEM (JEM‐1400FLASH). Briefly, RAW264.7 cells (1 × 10^6^ cells per well) were seeded in a 6‐well plate with 100 ng/mL LPS for 12 h plus 1 µM SIM. Then, the cells were collected, centrifuged, washed, and prefixed with 3% glutaraldehyde. Then, the tissue was postfixed in 1% osmium tetroxide, dehydrated in a series of acetone solutions, infiltrated in Epox 812 for a longer period, and embedded. The semithin sections were stained with methylene blue and ultrathin sections were cut with a diamond knife and stained with uranyl acetate and lead citrate. The sections were examined by TEM.

### 2.8 Reverse transcription‑quantitative polymerase chain reaction (RT‑qPCR)

Quantitative RT‐PCR was performed as described previously. RAW264.7 cells (10^5^ cells per well) were seeded in a 6sixwell plate and MCC950 (30 µM) was pretreated into LPS‐stimulated RAW264.7 cells in the presence or absence of SIM. Then, 100 ng/mL LPS was added to the culture medium and incubated for 12 h. Briefly, total RNA was extracted using TRIzol reagent (Invitrogen; Thermo Fisher Scientific Inc.) according to the manufacturer's instructions and quantified using a spectrophotometer set at 260 nm (Nanodrop, Thermo Fisher Scientific Inc.). Complementary DNA was synthesized from 1 μg of total RNA using reverse transcriptase from the PrimeScript RT reagent kit (Takara Bio, Inc.) RT‐qPCR was performed using the SYBR® Premix Ex Taq™ kit (TaKaRa Bio, Inc.). RT‐qPCR was performed using the CFX Connect Real‐Time PCR Detection System (Bio‐Rad). The thermocycling conditions for PCR were as follows: precycling at 95°C for 5 min, followed by 39 cycles of denaturation (95°C, 10 s) and annealing (60°C, 30 s). Relative target gene expression was normalized to that of GAPDH and calculated using the 2^−ΔΔCT^ method.^29^ The primer sequences used for this analysis are listed in Table 1 and all primer F and R were purchased from Sangon Biotech.

**Table 1:** Sequences of quantitative PCR primers.

Primers	Gene sequence
Mouse *CTSK* forward	5′‐GGAGGAAATGGCTGGACAC‐3′
Mouse *CTSK* reverse	5′‐CCAGGTTATGGGCAGAGA‐3′
Mouse *RANK* forward	5′‐TTCGACTGGTTCACTGCTCC‐3′
Mouse *RANK* reverse	5′‐TCAGGTGCTTTTCAGGGGAC‐3′
Mouse c*‐Fos* forward	5′‐CCGGTTCCTTCTATGCAGCA‐3′
Mouse *c‐Fos* reverse	5′‐GCTTGGGAAGGAGTCAGC‐3′
Mouse *GAPDH* forward	5′‐GGTTGTCTCCTGCGACTTCA‐3′
Mouse *GAPDH* reverse	5′‐TGGTCCAGGGTTTCTTACTCC‐3′

Abbreviations: CTSK, Cathepsin K; PCR, polymerase chain reaction; RANK, receptor activator of NF‐κB.

### 2.9 Western blot (WB) analysis

RAW264.7 cells (2 × 10^6^ cells per well) were seeded in a six‐well plate and incubated at 37°C overnight. After being treated with or without various concentrations SIM (0.1, 1, 5, and 10 µM) for 1 h, the cells were cultured with 100 ng/mL LPS for an additional 0, 6, 12 and 24 h. MCC950 (30 µM) was pretreated into LPS‐stimulated RAW264.7 cells in the presence or absence of 1 µM SIM for 6 h. Total protein was extracted from cultured cells using phenylmethylsulphonyl fluoride and RIPA buffer (Solarbio). A bicinchoninic acid protein assay kit (Solarbio) was used to measure the protein concentration according to the manufacturer's instructions. Equal amounts of protein (30 µg) from each sample were electrophoresed on 10% or 12% sodium dodecyl sulfate polyacrylamide gel electrophoresis and transferred to PVDF membranes. After being blocked in 5% skim milk at room temperature for 2 h, the membranes were incubated with primary antibodies at 4°C overnight, followed by HRP‐conjugated secondary antibodies at room temperature for 1.5 h. Primary antibodies against NLRP3, ATG7, P62, Beclin‐1, caspase‐1, cleaved‐caspase‐1, IL‐1β, TNF‐α, IL‐10, and LC3II were used to detect the expression of each protein, and *β‐actin* was used as a housekeeping gene. After being washed, the membranes were visualized with enhanced chemiluminescence solution (Millipore) and the bands were detected using the Gene Gnome Imaging System (Syngene). ImageJ software 6.0 was used to quantify images of the WB bands, which were normalized to that of the control.

### 2.10 Statistical analysis

Statistical analysis was performed by GraphPad Prism 9.0 statistical software. Normal distribution of the data was confirmed with the Shapiro‒Wilk normality test. One‑way analysis of variance followed by Tukey's posthoc test was used for multiple comparisons. Each experiment was repeated three times and all quantitative data are presented as the mean ± SD. *p* < .05 was considered to indicate statistical significance.

## 3 RESULTS

### 3.1 SIM reduced LPS‑induced inflammatory level in the pre‐OCs

**Figure 1:**
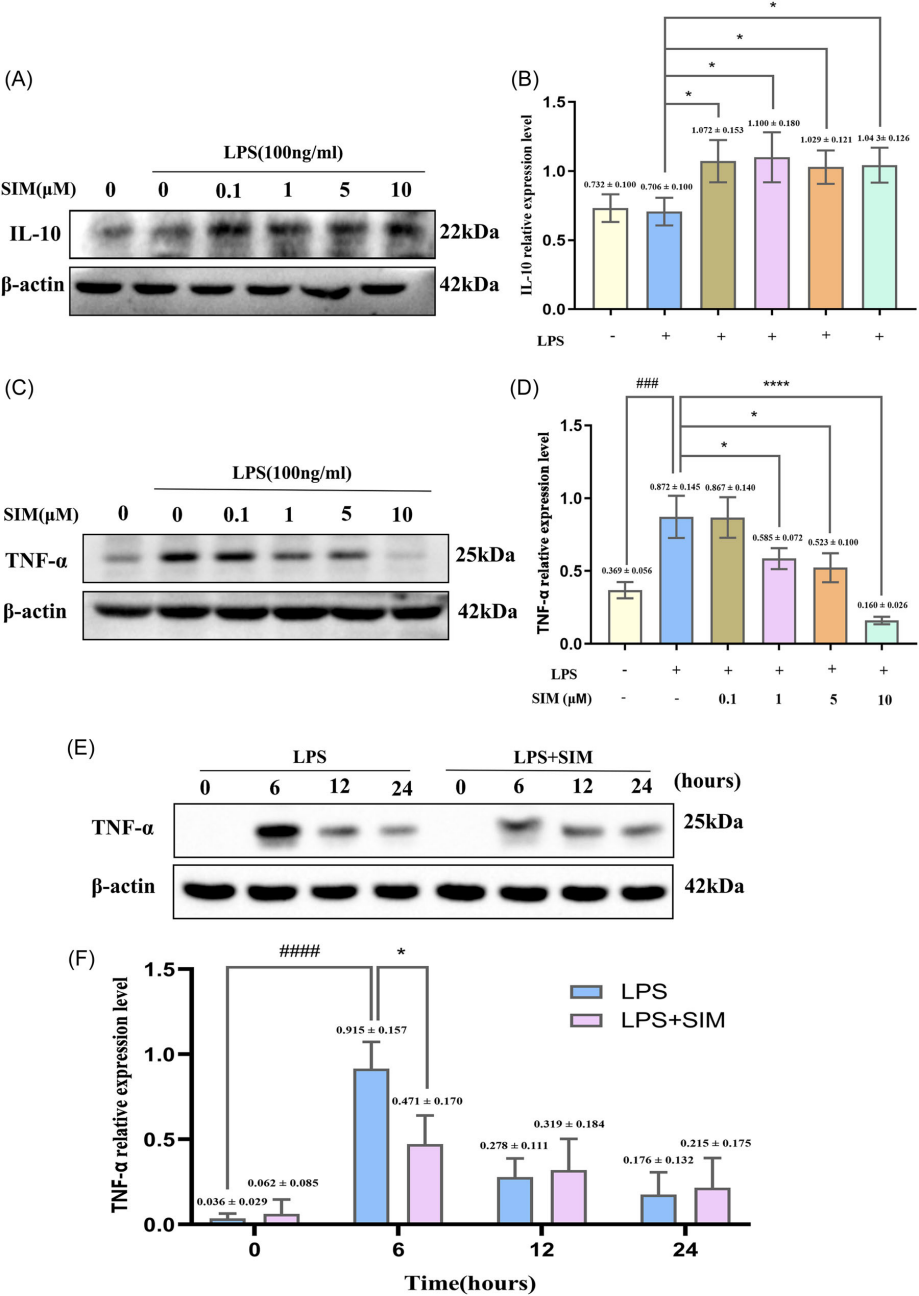
Simvastatin (SIM) reduced induced lipopolysaccharide (LPS)‐induced inflammatory level. To explore the peak time of inflammation induced by LPS and the optional concentration of anti‐inflammation of SIM, western blot (WB) analysis was performed using tumor necrosis factor‐α (TNF‐α) and Interleukin‐10 (IL‐10) antibodies. RAW264.7 cells were cultured with 0.1, 1, 5, and 10 μM SIM for 1 h and then treated them with 100 ng/mL of LPS for 6 h (A, C) and were induced with LPS for the indicated time points following pre treatment with 1 μM SIM for 1 h (E). The band intensities were quantified using Image J software (B, D, F). Bars represent the mean ± SD of three independent experiments. ###*p* < .001 and ####*p* < .0001 compared with control group; *p < .05 and ****p < .0001 compared with LPS‐treated group.

To detect the peak time of inflammation induced by LPS and the optional concentration of anti‐inflammation of SIM, the cells were stimulated with LPS and treated with 0.1, 1, 5, and 10 µM SIM for different time points (0, 6, 12, 24 h). WB results showed that 1, 5, and 10 µM SIM significantly suppressed LPS‐induced inflammatory cytokine TNF‐α expression while promoted anti‐inflammatory cytokine IL‐10 expression, especially at 6 h (Figure 1A–F).

### 3.2 SIM inhibited LPS‑induced osteoclastogenesis

**Figure 2:**
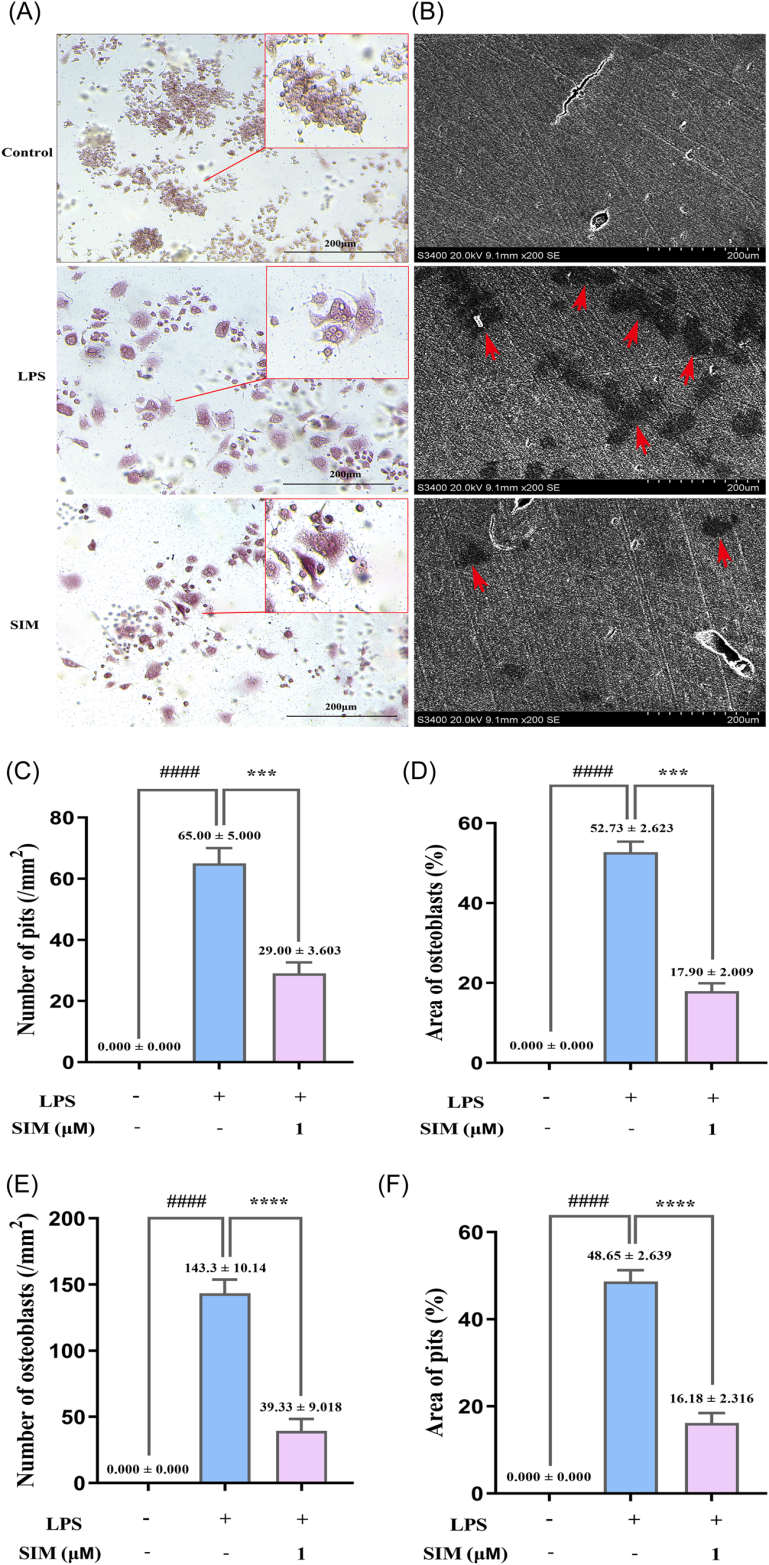
Simvastatin (SIM) inhibited the lipopolysaccharide (LPS)‑induced osteoclast (OC) differentiation and function. (A, B) RAW264.7 cells were treated with 1 µM SIM in the presence or absence of 100 ng/ml LPS for 3 days (tartrate‐resistant acid phosphatase [TRAP] staining) or 10 days (Resorption pit assay). Bone resorption pits (red arrows) were visualized under a scanning electron microscope. (C–F) The number and aera percentage of OCs and pits were counted per field of microscope. Bars represent the mean ± SD of three independent experiments. ####*p* < .0001 compared with control group; ***p < .001 and ****p < .0001 compared with LPS‐treated group.

To examine whether SIM inhibits LPS‐induced OC differentiation, RAW264.7 cells were treated with 1 µM SIM in the presence of LPS. TRAP staining showed that LPS could stimulate the differentiation of RAW264.7 cells into OCs, and the fusion of multiple monocytes into single multinucleated OCs was observed at day 3. However, the number of TRAP‐positive cells and the percentage of the OC area were significantly decreased in the 1 µM SIM group. (Figure 2A,C,D) Therefore, these results suggest that SIM inhibits OC differentiation induced by LPS.

### 3.3 SIM inhibited LPS‑induced OC formation

**Figure 3:**
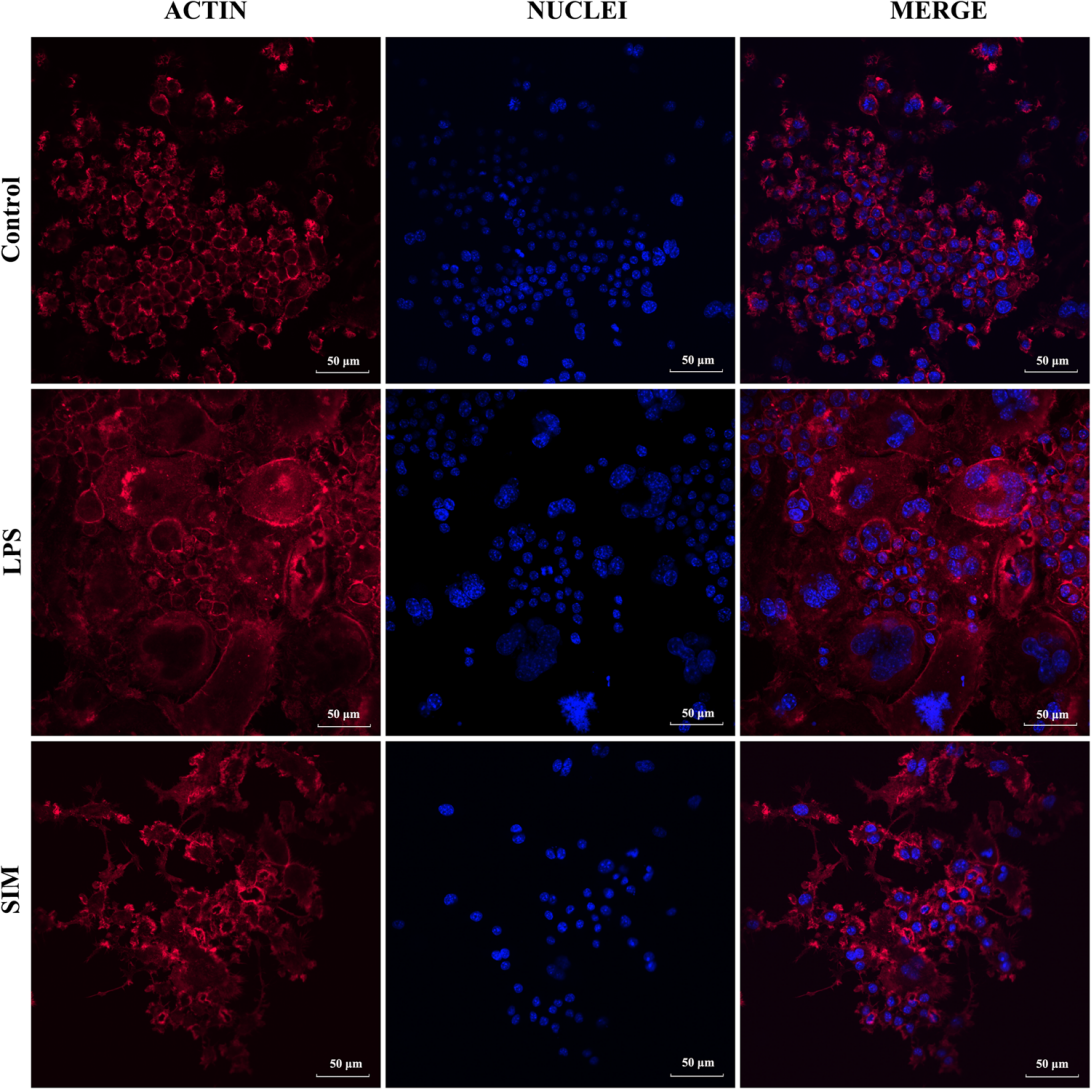
Simvastatin (SIM) inhibited the lipopolysaccharide (LPS)‑induced osteoclast (OC) formation. RAW264.7 cells were treated with 1 µM SIM in the presence or absence of 100 ng/ml LPS for 3 days until mature OCs were observed. Cell nuclei and F‐actin rings were stained with 4′,6‐diamidino‐2‐phenylindole (DAPI) and TRITC phalloidin, respectively. Fluorescence was detected by using a confocal microscope.

To investigate OC formation, the cells were stimulated with LPS and treated with 1 µM SIM for 5 days. Mature OCs containing actin ring structures are a prerequisite for OC bone resorption. Similarly, we performed actin ring and DAPI staining and showed that well‐structured actin rings were observed in LPS‐induced OCs. However, the formation of actin ring structures in LPS‐induced OCs appeared abnormal or immature, and the number of OCs was decreased in the 1 µM SIM group (Figure 3).

### 3.4 SIM attenuated LPS‑induced OC function

Then, we investigated whether SIM modulates mature OC activity by performing a resorption pit assay. RAW264.7 cells were plated on bone slices, which were treated with various concentrations of SIM in the presence or absence of 100 ng/ml LPS. The results indicated that the area of the OC bone resorption pits was markedly decreased by SIM at an appropriate concentration compared with that in the SIM‐free group. Furthermore, almost no resorption pits were observed in the groups that were treated with 1 µM SIM (Figure 2B,E,F). These results suggest that treatment with SIM markedly attenuates the bone‐resorption activity of OCs. This may, at least partially, be explained by the ability of SIM to impair osteoclastogenesis.

### 3.5 SIM suppressed LPS‐induced OC‐associated gene expression

**Figure 4:**
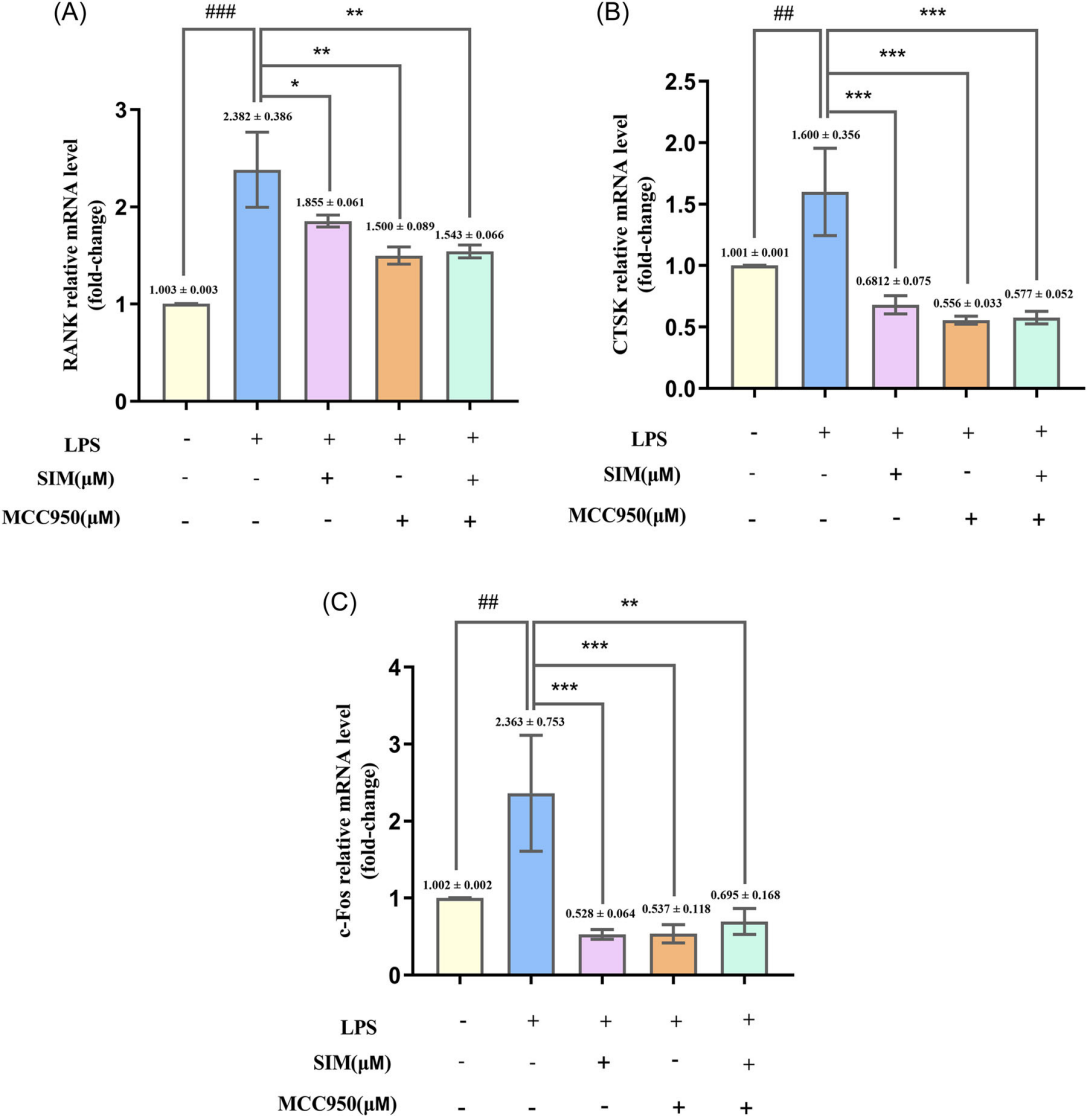
Simvastatin (SIM) suppresses lipopolysaccharide (LPS)‐induced osteoclast (OC)‐associated gene expression. RAW264.7 cells were stimulated with NOD‐like receptor family pyrin Domain‐containing protein 3 (NLRP3) inhibitor MCC950 (30 µM) and SIM, then treated them with 100 ng/mL of LPS for 12 h. (A–C) The expression of OC‐specific genes (*RANK*, *CTSK*, and *c‐Fos*) were detected by reverse transcription‑quantitative polymerase chain reaction (RT‐qPCR). Bars represent the mean ± SD of three independent experiments. ##*p* < .01 and ###*p* < .001 compared with control group; *p < .05, **p < .01, and ***p < .001 compared with LPS‐treated group.

To further explore the role of SIM in OC differentiation, we analyzed the messenger RNA (mRNA) expression levels of LPS‐induced OC‐associated genes in the absence or presence of SIM with MCC950 for 12 h. The mRNA expression levels of receptor activator of NF‐κB (RANK), Cathepsin K (CTSK) and c‐Fos were markedly enhanced by stimulation with LPS. In contrast, treatment with SIM after 12 h of LPS stimulation markedly suppressed the mRNA expression of these genes, and there was no significant difference between the MCC950 and SIM groups (Figure 4).

### 3.6 SIM attenuated LPS‐induced autophagy in pre‐OCs and suppressed the NLRP3 signaling pathway

**Figure 5:**
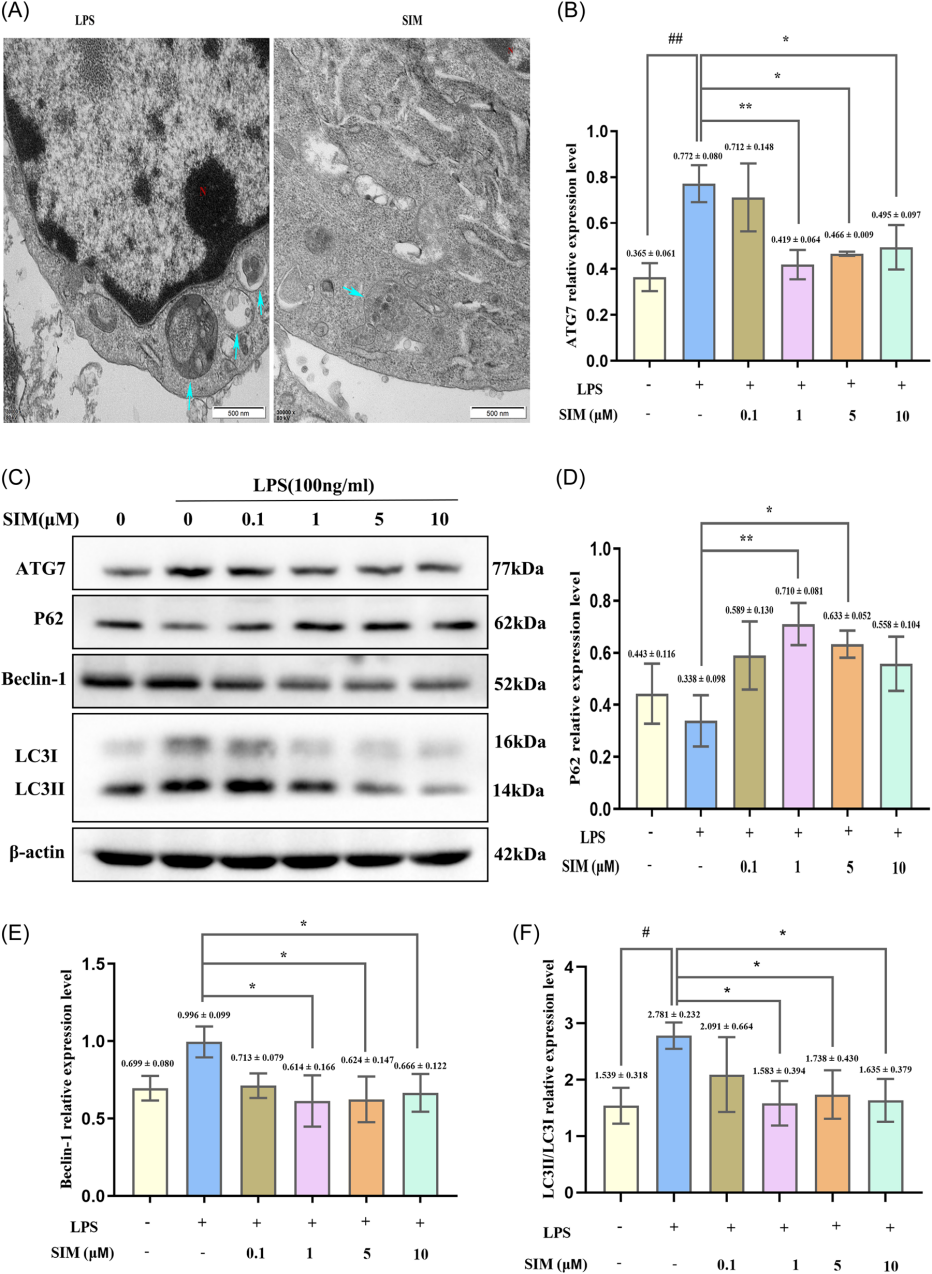
Simvastatin (SIM) attenuated lipopolysaccharide (LPS)‐induced autophagy in pre‐osteoclasts (OCs). (A) RAW264.7 cells were cultured with 100 ng/mL of LPS for 12 h plus 1 µM SIM, then autophagosomes (blue arrow) as observed by transmission electron microscopy (TEM), *N* represents the nucleus. (C) RAW264.7 cells were incubated with 0.1, 1, 5, or 10 µM SIM for 1 h and then treated them with 100 ng/mL of LPS for 6 h, then western blot (WB) analysis was performed using autophagy‐related molecules. (B, D–F) The band intensities were quantified using ImageJ software. Bars represent the mean ± SD of three independent experiments. ##*p* < .01 compared with control group; *p < .05, **p < .01, and ***p < .001 compared with LPS‐treated group.

**Figure 6:**
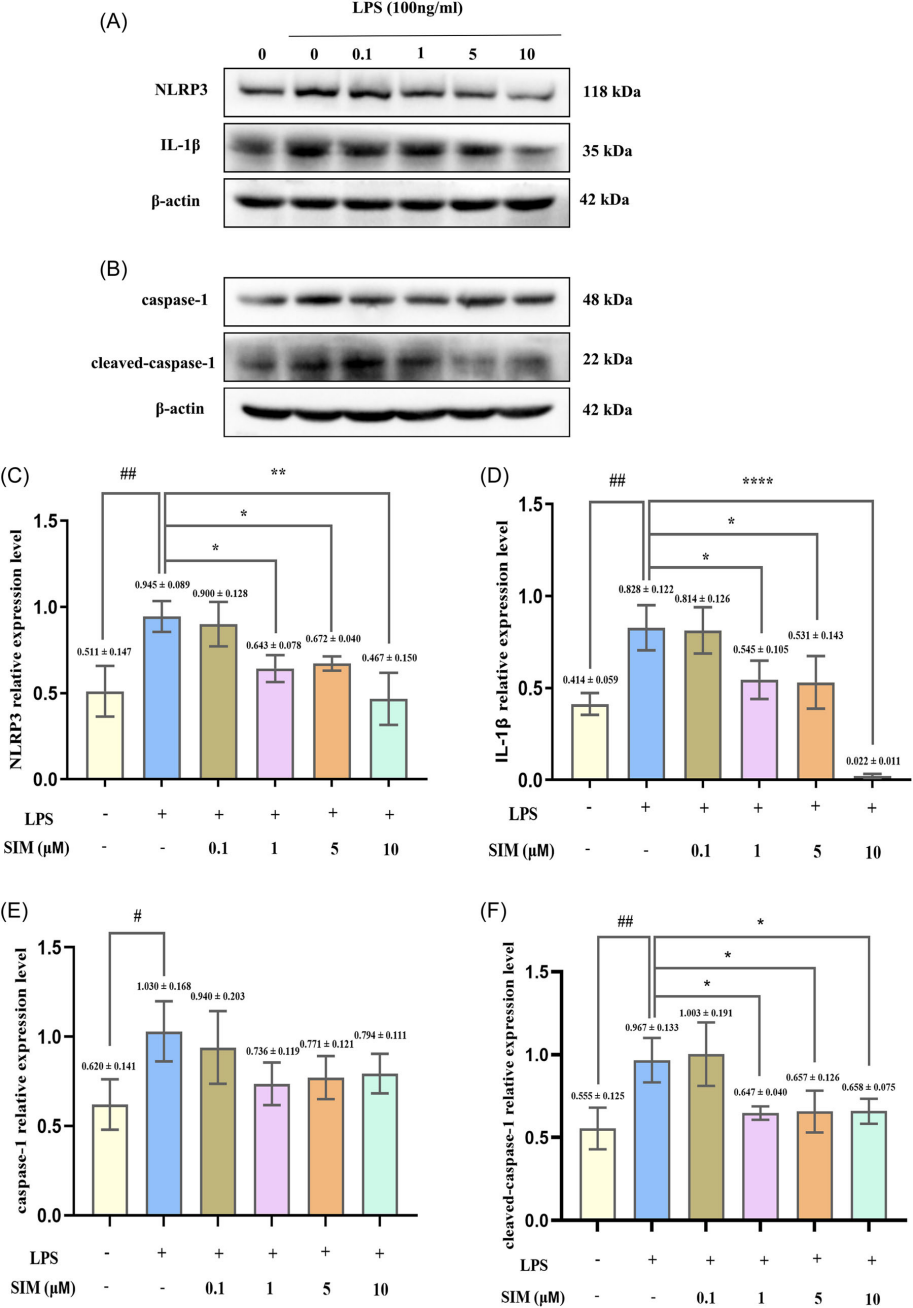
Simvastatin (SIM) suppressed the expression of NOD‐like receptor family pyrin Domain‐containing protein 3 (NLRP3)‐related proteins. Western blot (WB) analysis was performed using NLRP3 related antibodies. (A,B) RAW264.7 cells were cultured with 0.1, 1, 5, and 10 µM SIM for 1 h and then treated them with 100 ng/mL of lipopolysaccharide (LPS) for 6 h. (C–F) The band intensities were quantified using ImageJ software. Bars represent the mean ± SD of three independent experiments. #*p* < .05 and ##*p* < .01 compared with control group; *p < .05, **p < .01, and ****p < .0001 compared with LPS‐treated group.

**Figure 7:**
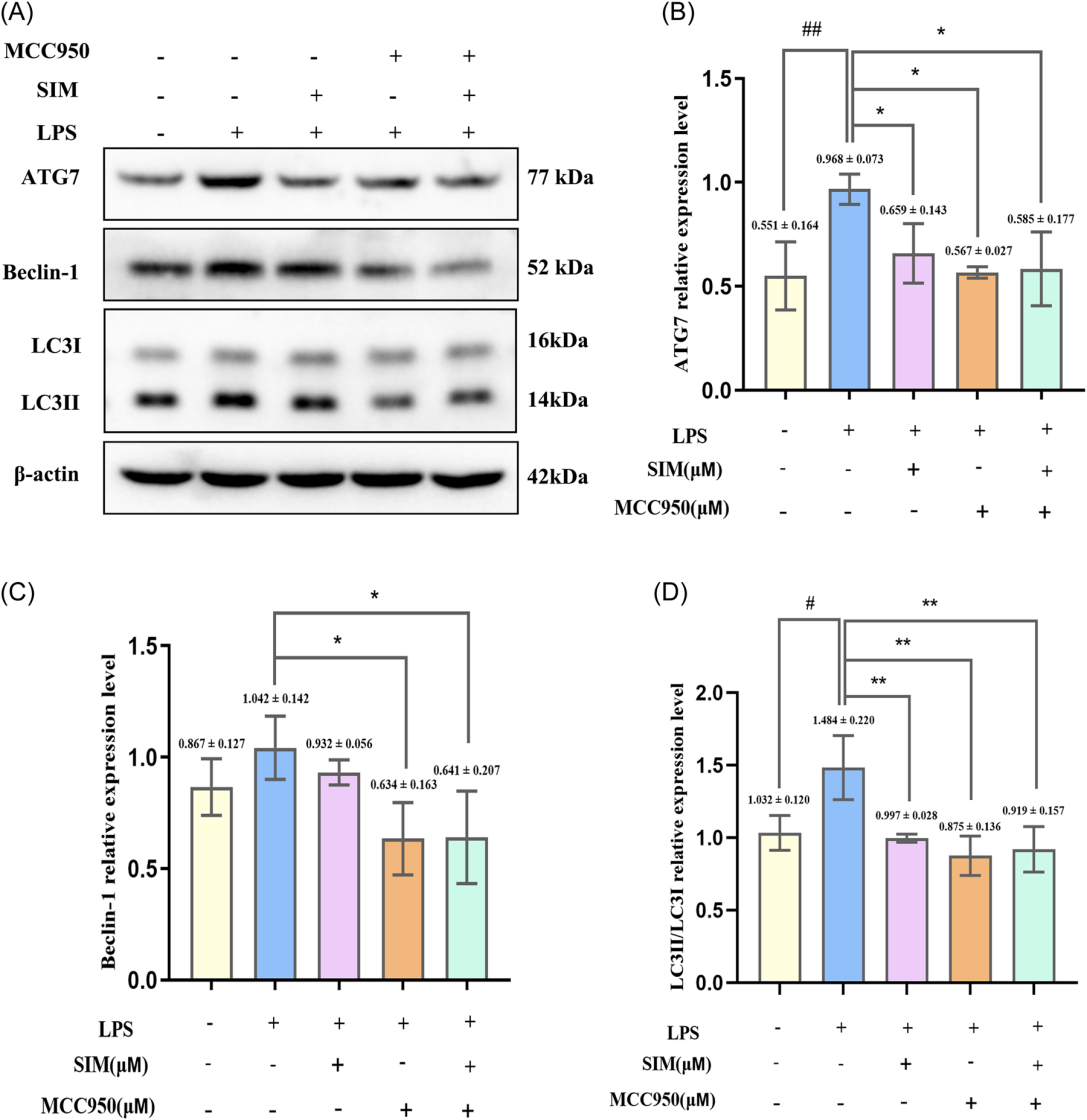
MCC950 is involved in the inhibition of Simvastatin (SIM) on autophagy‐related proteins in lipopolysaccharide (LPS)‐treated RAW264.7 macrophages. (A) MCC950 was pretreated into LPS‐stimulated RAW264.7 macrophages in the presence or absence of SIM. (B–D) The band intensities were quantified using ImageJ software. Bars represent the mean ± SD of three independent experiments. #*p* < .05 and ##*p* < .01 compared with control group; *p < .05 and **p < .01 compared with LPS‐treated group.

As mentioned in the Section 1, autophagy and NLRP3 signaling pathway are closely related in inflammatory bone microenvironment. Therefore, to determine the underlying mechanism of inflammatory osteoclastogenesis inhibited by SIM, we explored the role of autophagy in SIM mediated anti‐inflammatory osteoclastogenesis through NLRP3 signaling pathway. Compared with the LPS group, the TEM results showed a significant reduction in autophagosomes in the 1 µM SIM group (Figure 5A). Moreover, after treatment with different concentrations of SIM (0.1, 1, 5, or 10 µM) and 100 ng/mL LPS for 6 h, the expression levels of autophagy‐related proteins and NLRP3‐related proteins were detected by WB analysis (Figures 5C and 6A,B). Compared with the control group, there was no significant increase in the expression of Beclin‐1 and p62 proteins in the LPS group (Figure 5D,E). However, compared to the LPS group, the protein expression levels of LC3II, Beclin‐1, ATG7, NLRP3, cleaved‐caspase‐1 and IL‐1β in the 1, 5, and 10 µM SIM groups were significantly decreased, while the expression of p62 was significantly increased in the 1 and 5 µM SIM groups (Figure 5B,D–F and 6C–F). Most importantly, MCC950 also suppressed autophagy related proteins expression, especially ATG7 and LC3II. And there was no significant difference between the MCC950 and SIM groups (Figure 7A–D).

## 4 DISCUSSION

In the present study, we found that SIM inhibited the LPS‐induced OC differentiation, formation, function and OC‐associated genes expression. Moreover, autophagy and NLRP3 signaling pathway are involved in this process. The NLRP3 inflammasome has been reported to be associated with the pathogenesis of inflammatory bone diseases.^30^ Furthermore, a large number of studies have shown that there is an interaction between the NLRP3 inflammasome and autophagy, and activation of the NLRP3 inflammasome can regulate the induction of autophagy, while autophagy can regulate inflammasome activation and inhibit inflammasome activity.^21^, ^31^, ^32^, ^33^ It has been shown that SIM is found to suppress osteoclastic differentiation in bone tissue^19^ and promote osseointegration around implants in animal studies.^20^ However, there was no study to investigate the underlying mechanisms of SIM on LPS‐induced OC formation and bone resorption. Studies have suggested that reducing the number and activity of OCs by regulating LPS‐induced pre‐OC autophagy is a potential therapeutic strategy for the treatment of inflammatory bone loss.^34^ Therefore, it is worth studying the influence of SIM on LPS‐induced inflammation and autophagy in pr‐OCs. According to the results, we found that SIM could attenuate autophagy to inhibit LPS‐induced osteoclastogenesis by suppressing the NLRP3‐mediated inflammatory response in pre‐osteoclastic RAW264.7 cells.

OCs induced by RANKL are often used to simulate bone destructive diseases, while OCs induced by LPS are often used to simulate chronic bone infectious diseases. Related studies have been reported that LPS‐activated RAW264.7 cell fusion without the assistance of other cells and multinucleated osteoclastic RAW‐OC formation in a RANKL‐independent manner.^35^, ^36^, ^37^ In addition, small multinucleated OCs derived from RAW264.7 cells form within 16 h after LPS induction,^38^ and our result of TNF‐a protein (Figure 1C) was also consistent with previous study,^24^ suggesting that 6 h of LPS‐induced inflammation caused the greatest change in related protein levels, so we studied the effect of short‐term induction of LPS at the molecular level. Based on cell morphology experiments, we observed that the number of TRAP‐positive OCs decreased significantly, the morphology of OCs appeared abnormal or immature, and the bone resorption activity of OCs was weakened in the group that were treated with 1 µM SIM. These observations were in line with the report from Jin et al. that SIM could inhibit lipopolysaccharide‐induced osteoclastogenesis in vivo experiment,^39^ but few studies have been reported in vitro. Our results also showed that SIM significantly suppressed LPS‐induced inflammatory cytokine TNF‐α expression while promoted anti‐inflammatory cytokine IL‐10 expression in a dose‐independent manner. TNF‐α is believed to be the main factor that mediates LPS‐induced OC formation,^40^, ^41^ whereas IL‐10 inhibits OC differentiation and osteolysis.^42^ It has been demonstrated that statins inhibit production of pro‐inflammatory mediators in LPS‐stimulated RAW264.7 cells.^43^, ^44^ SIM in peripheral blood mononuclear cells had no dose‐dependent effect and low concentration (10^−8^ M) SIM also promoted the secretion of IL‐10, which was consistent with the results of our experiment. And there was an imbalance between pro‐ and anti‐inflammatory cytokine productions in mononuclear cells under inflammatory environmental.^45^ Moreover, the lower concentration of SIM promotes the expression of anti‐inflammation related proteins in a short time, but this effect of is not obvious with the extension of time. Therefore, our experimental results revealed that 0.1 µM SIM was only effective in increasing the expression of anti‐inflammatory cytokine IL‐10.

At the genetic level, LPS induced OC‐related genes *RANK*, *CTSK*, and *c‐Fos* expression, which presumably promote differentiation and maturation of OCs, and then cause inflammatory bone resorption.^46^, ^47^, ^48^ We have also observed that SIM suppresses LPS‐caused increase in RANK, CTSK and c‐Fos levels, which indicated that SIM inhibited the differentiation and formation of OCs.^39^, ^49^ As for RANK was activated without RANKL in Figure 4, it is because the macrophage RAW264.7 has been induced into OC precursors or partial OCs by LPS, and RANK is highly expressed on the surface of OC precursors and OCs.

Then, we focused on the effects of SIM on autophagy and NLRP3 activation at the protein level. In this study, we found that after 6 h of LPS stimulation, NLRP3, caspase‐1, IL‐1β, ATG7, and LC3II protein levels were significantly upregulated, whereas the expression of P62 was decreased, indicating that the LPS‐mediated NLRP3 signaling pathway and autophagy are both activated. It has been reported that LPS‐induced autophagy is responsible for increasing the number and activity of OCs in inflammatory bone loss.^32^ During the process of bone remodeling, the activation of autophagy factors in OCs, including Beclin1, LC3, ATG5, and ATG7, can promote OCs to form folded edges, secrete factors, and cause bone resorption.^24^, ^50^ As for the expression of P62 and Beclin‐1 proteins was no significant difference between control group and LPS‐treated group, which may be due to the fact that we starved the cells for a short time (4 h) before adding drugs. The hungry cells can not only absorb drugs better, but also induce autophagy. As a result, the expression of autophagy protein increased in some controls. However, the other autophagy proteins in the control group had no significant changes, which may be related to the short time of starvation. Furthermore, 1 and 5 µM SIM could increase p62 levels, whereas 10 µM SIM was ineffective, it may be that p62 protein expression in a dose‐dependent manner in 0.1–5 µM, whereas 10 µM SIM suppresses p62 expression. Among the pro‐inflammatory cytokines, IL‐1β strongly induces bone destruction by promoting OC differentiation and activity. IL‐1β is one of the primary members of the IL‐1 family and plays an important role in bone loss following inflammatory infection.^51^ In general, LPS stimulates the secretion of various cytokines in the microenvironment, including IL‐1β and TNF‐α, which are involved in LPS‐mediated bone resorption. However, compared with the LPS group, these events were inhibited by SIM, which indicated that SIM inhibited autophagy‐related proteins and NLRP3‐related proteins expression did not increase in a time‐dependent manner but did fluctuate, thereby decreasing the high levels of autophagy could be a potential strategy for treating and/or preventing inflammation‐related diseases,^52^ including inflammatory bone resorption. However, this finding was also inconsistent with some previous literature reports which believed that it was to attenuate the level of inflammation by promoting autophagy, thereby alleviating the development of the inflammation‐related disease.^53^, ^54^ Subsequently, the WB results of NLRP3 inhibitor MCC950 clearly showed that SIM inhibited the high expression of autophagy protein in inflammatory state, and there was no significant difference in autophagy level between MCC950 group and SIM + MCC950 group, indicating that SIM and MCC950 had no synergistic effect. However, as for treatments with SIM alone failed to suppress the increase of Beclin‐1, it may be that compared with MCC950 alone group and SIM + MCC950 group, the inhibitory effect of SIM is not so obvious for this protein. Taken together, these results indicated that SIM could down‐regulate overactive autophagy to alleviate the further development of inflammation. However, we used simple culture induction of RAW264.7 macrophages instead of coculture with MC3T3 embryonic osteoblasts, so these studies did not address the effect of SIM on bone formation under the influence of LPS and the effect of the interaction between SIM, LPS and autophagy on osteoblastogenesis remains largely unknown. Moreover, further investigation is needed to explore the direct connection between SIM, LPS and autophagy in vivo.

In conclusion, in this study, SIM may reduce autophagy secretion to attenuate LPS‐induced osteoclastogenesis through the NLRP3 signaling pathway. Our findings provide a new perspective into the mechanisms underlying inflammatory responses induced by LPS. These findings indicate that therapeutic strategies targeting autophagy may provide a new method for the treatment of inflammatory diseases. Therefore, our research may provide a reference for evaluating the pharmacological effect of these statin products on bone tissue in an inflammatory state, as well as a direction for the application of these drugs in the regulation of bone metabolism in infectious bone disease.

## AUTHOR CONTRIBUTIONS

Jian Liao conceived and designed the research. Yuting Cheng, Wenjun Jin, Lin Zheng, Xiaolin Huang, and Shanshan Luo performed the experiments. Yuting Cheng performed preliminary data analysis and wrote manuscript outline. Chidchanok Leethanakul and Bancha Samruajbenjakun supervised the investigation and proof checked data for error. The article was guided and revised by Jian Liao, and Wei Hong, Yuting Cheng, and Jian Liao made equal contribution to this research as co‐first authors. All authors have read and approved the final manuscript.

## CONFLICT OF INTEREST STATEMENT

The authors declare no conflict of interest.

## ETHICS STATEMENT

Not applicable.

## Data Availability

The datasets generated during and analyzed during the current study are available from the corresponding author on reasonable request.
